# Defining the seasonality of respiratory syncytial virus around the world: National and subnational surveillance data from 12 countries

**DOI:** 10.1111/irv.12885

**Published:** 2021-07-13

**Authors:** Lisa Staadegaard, Saverio Caini, Sonam Wangchuk, Binay Thapa, Walquiria Aparecida Ferreira de Almeida, Felipe Cotrim de Carvalho, Rodrigo A. Fasce, Patricia Bustos, Jan Kyncl, Ludmila Novakova, Alfredo Bruno Caicedo, Domenica Joseth de Mora Coloma, Adam Meijer, Mariëtte Hooiveld, Q. Sue Huang, Tim Wood, Raquel Guiomar, Ana Paula Rodrigues, Vernon Jian Ming Lee, Li Wei Ang, Cheryl Cohen, Jocelyn Moyes, Amparo Larrauri, Concepción Delgado‐Sanz, Clarisse Demont, Mathieu Bangert, Michel Dückers, Jojanneke van Summeren, John Paget

**Affiliations:** ^1^ Nivel (Netherlands Institute for Health Services Research) Utrecht The Netherlands; ^2^ Royal Centre for Disease Control Ministry of Health Thimphu Bhutan; ^3^ Department of Immunization and Communicable Diseases Ministry of Health Brasilia Brazil; ^4^ Subdepartamento Enfermedades Virales Instituto de Salud Pública de Chile Santiago Chile; ^5^ Sección Virus Respiratorios, Subdepartamento Enfermedades Virales Instituto de Salud Publica de Chile Santiago Chile; ^6^ Department of Infectious Diseases Epidemiology National Institute of Public Health Prague Czech Republic; ^7^ Department of Epidemiology and Biostatistics, Third Faculty of Medicine Charles University Prague Czech Republic; ^8^ National Reference Laboratory for Influenza and Other Respiratory Viruses National Institute of Public Health Prague Czech Republic; ^9^ Universidad Agraria del Ecuador Guayaquil Ecuador; ^10^ Instituto Nacional de Investigación en Salud Pública (INSPI) Centro de Referencia Nacional de Influenza y otros Virus Respiratorios Guayaquil Ecuador; ^11^ National Institute for Public Health and the Environment Bilthoven The Netherlands; ^12^ Institute of Environmental Science and Research Limited (ESR) National Centre for Biosecurity and Infectious Disease (NCBID) Upper Hutt New Zealand; ^13^ Instituto Nacional de Saúde Doutor Ricardo Jorge Lisbon Portugal; ^14^ Ministry of Health Singapore; ^15^ Saw Swee Hock School of Public Health Singapore; ^16^ National Centre for Infectious Diseases Singapore; ^17^ Centre for Respiratory Disease and Meningitis National Institute for Communicable Diseases Johannesburg South Africa; ^18^ School of Public Health University of Witwatersrand Johannesburg South Africa; ^19^ National Centre of Epidemiology, CIBER Epidemiología y Salud Pública (CIBERESP) Institute of Health Carlos III (ISCIII) Madrid Spain; ^20^ Sanofi Pasteur Lyon France

**Keywords:** epidemiology, respiratory syncytial virus, seasonality, surveillance

## Abstract

**Background:**

Respiratory syncytial virus (RSV) infections are one of the leading causes of lower respiratory tract infections and have a major burden on society. For prevention and control to be deployed effectively, an improved understanding of the seasonality of RSV is necessary.

**Objectives:**

The main objective of this study was to contribute to a better understanding of RSV seasonality by examining the GERi multi‐country surveillance dataset.

**Methods:**

RSV seasons were included in the analysis if they contained ≥100 cases. Seasonality was determined using the “average annual percentage” method. Analyses were performed at a subnational level for the United States and Brazil.

**Results:**

We included 601 425 RSV cases from 12 countries. Most temperate countries experienced RSV epidemics in the winter, with a median duration of 10–21 weeks. Not all epidemics fit this pattern in a consistent manner, with some occurring later or in an irregular manner. More variation in timing was observed in (sub)tropical countries, and we found substantial differences in seasonality at a subnational level. No association was found between the timing of the epidemic and the dominant RSV subtype.

**Conclusions:**

Our findings suggest that geographical location or climatic characteristics cannot be used as a definitive predictor for the timing of RSV epidemics and highlight the need for (sub)national data collection and analysis.

## 1 INTRODUCTION

Respiratory syncytial virus (RSV) infections are one of the leading causes of lower respiratory tract infections (LRTI).^1^, ^2^ While the burden is highest in children, RSV infections are also associated with high rates of hospitalization and morbidity in the elderly (≥65 years) and high‐risk adults.^3^, ^4^ With no existing vaccine, prevention methods include hand washing, avoiding contacts with those infected with RSV, and passive immunization through the administration of the monoclonal antibody (mAb)—palivizumab.^5^ The latter, requiring monthly administration during the RSV season, is recommended in the first year of life in high‐risk infants.^5^ For prevention methods to be deployed effectively and to improve the planning of health services, an improved understanding of the seasonality of RSV is required.

The lack of global surveillance data results in few studies exploring the regional or global RSV seasonality using a multicountry dataset and uniform method. Studies that do transcend the national level are limited to reviews or meta‐analyses based on previously published national seasonality estimates,^6^, ^7^, ^8^ and few examples exist that are based on surveillance data.^9^, ^10^, ^11^, ^12^, ^13^, ^14^, ^15^ Importantly, the range of statistical methods used to define seasonality in the literature hampers a robust comparison across studies.

Previous studies have found that the seasonality of RSV in temperate climates is stable over time, with most countries experiencing a distinct peak in RSV cases per season. Temperate countries in the Northern Hemisphere generally experience the start of the RSV season between September and January and those in the Southern Hemisphere between March and June, closely aligned with colder temperatures.^7^ The seasonality of RSV in (sub)tropical climates appears less consistent—some studies indicate that the peak of RSV epidemics is closely aligned with the rainy season,^6^, ^10^ and others have found increased RSV activity with higher temperatures.^16^, ^17^, ^18^ Additionally, studies have explored the impact of RSV subtype dominance on seasonality, but the association between an RSV A or B dominant season and the timing of epidemics remains unclear.^19^, ^20^, ^21^ Several (other) climatic associations have been sought to explain variation within climate zones, but a clear predictor of seasonality has yet to be established.

The Global Epidemiology of RSV in Hospitalized and Community care (GERi) study collects detailed surveillance data to better describe the epidemiology of RSV and support prevention and control measures. Here, we aim to describe the start, end, peak, and duration of RSV epidemics in 12 countries around the world. We also assess the impact of RSV subtype dominance on the timing of RSV seasonality.

## 2 METHODS

### 2.1 Global RSV virological surveillance data

The GERi network, consisting of 16 countries, and the surveillance systems from which the virological data have emerged have been described previously.^22^ Briefly, data were stratified by level of care (hospitalized and community) and both case definitions (ILI at community level and SARI at hospitalized level), and diagnostic methods were largely similar across participating countries. For the current analysis, seasons containing <100 cases at national level were excluded. Large countries for which we had subnational data (Brazil and the United States) were considered as separate areas, and seasonality was assessed at a subnational level.

### 2.2 Geographic and seasonal classification

Countries or regions were considered to be either temperate (latitude < −23.5° or >23.5°) or (sub)tropical (latitude between −23.5° and 23.5°).^13^ Seasons in the Northern Hemisphere, experiencing a temperate climate (latitude > 23.5°), were defined as ranging from Week 27 to Week 26 of the following year, whereas the rest of the seasons were defined as ranging from Week 1 to Week 52 of the same calendar year. For seasons including a 53rd week, weeks were pulled backwards omitting 1 week with no cases.

### 2.3 Average annual percentage

To estimate the start and end of the epidemic, we calculated the “average annual percentage” (*AAP*) of cases for each week.^13^, ^23^ The *AAP* was calculated as follows:

$$\mathrm{AA}P_{i}=\frac{n_{i}}{\sum_{1}^{x}n_{i}}*100$$

where *i* denotes the week, *n* denotes the number of cases, and *x* is the total number of weeks in a given season. Weeks were then sorted in descending order, and the first weeks to add up to at least 75% of the *AAP* were identified as *epidemic weeks*. To identify the start and end of an epidemic, the *epidemic weeks* were sorted by time. The start and end of the epidemic were then identified as the first and last week of the longest consecutive *epidemic weeks* with a 2‐week gap allowed. The number of weeks from the start to end of the epidemic was defined as the duration.

If multiple weeks had the same *AAP*_*i*_—and not all of these were required to make up 75% of the *AAP*—only those adjacent to the epidemic were included as *epidemic weeks*. The peak week was defined as the week with the majority of cases—if there were multiple weeks with the same number of cases, a 3‐week average was calculated with these weeks as the central week.^23^ The central week with the highest 3‐week average was then defined as the peak week.

### 2.4 RSV subtype classification

The occurrence of RSV A or B dominant seasons was explored using both a ≥60% or ≥70% threshold to define a subtype as being dominant. Seasons were excluded if <100 cases were subtyped. We investigated whether there were differences in the start, end, and duration of RSV A or B dominant epidemics using a regression analysis with robust standard errors, which took into account the potential clustering of individual country results.

## 3 RESULTS

After exclusion, the analysis included 12 countries and 210 seasons between 2000 and 2020 (Cameroon, Romania, Vietnam, and Russia were excluded), in both the Northern and Southern Hemispheres (Table S1). This included 131 seasons on a subnational level. The dataset consisted of 501 425 cases of RSV with a median of 1585 cases per country, region, and season. The median, end, start, peak, and duration of RSV epidemics are summarized by hemisphere, climate zone, and country in Table S1.

### 3.1 Temperate climate zones

**FIGURE 1:**
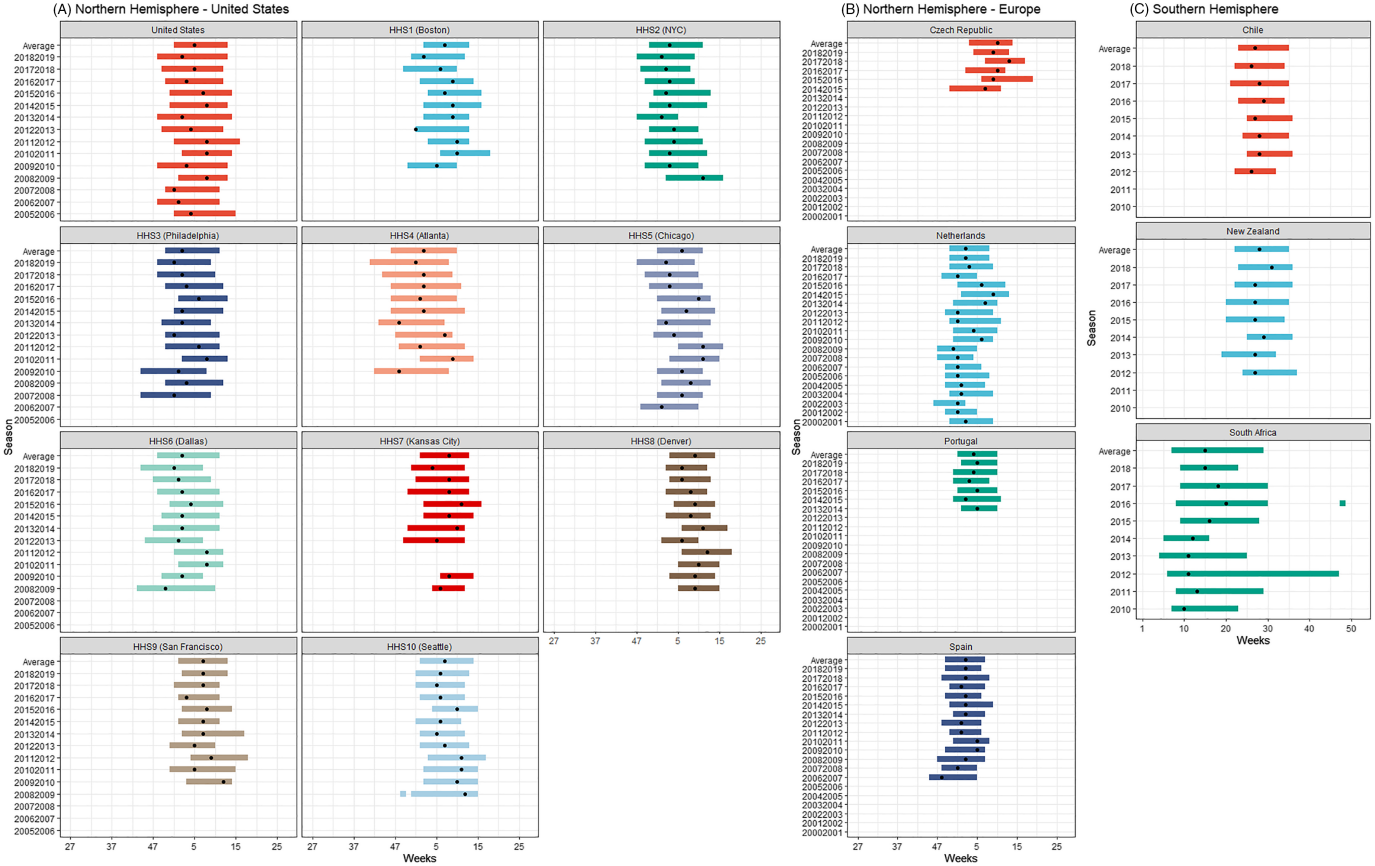
(A–C) RSV seasonality per country (or subnational level) and season in countries experiencing a temperate climate. *Dot indicates peak of the epidemic*

In countries in the Northern Hemisphere experiencing a temperate climate, the median start of epidemics consistently occurred in December or January and ended in February or March (Figure 1A,B and Table S1). This resulted in a median epidemic duration of 10 to 17 weeks (2–5 months). In Europe, most countries showed stable seasonality both nationally and comparatively, with all epidemics in the Netherlands, Spain, and Portugal starting in December and a median epidemic duration of 10 or 11 weeks. The same holds true for the RSV epidemics in the United States on a national level, which started in December and ended in March. The exception to this general pattern was the Czech Republic, where the median epidemic start week was Week 3 (January) and there was an alternating start date from 1 year to another, with a slightly earlier start (December or January) for the 2014/2015, 2016/2017, and 2018/2019 seasons and a later start (February) for the 2015/2016 and 2017/2018 seasons (Figure 1B).

For the Southern Hemisphere, both Chile and New Zealand, two countries which (for large parts) experience a temperate climate, also showed stable seasonal RSV epidemics (Figure 1C). Both countries experienced an RSV epidemic starting between Weeks 19 and 25 (May or June), ending between Weeks 32 and 37 (August or September), and a peak between Weeks 26 and 31 (June or August), which is largely consistent with the coldest months. The duration of these epidemics was anywhere between 11 and 15 weeks (2.5–3.5 months). In contrast, the RSV epidemics in South Africa were different as the seasons started earlier—between Weeks 4 and 9 (January or February)—and showed more variability in the end and duration from one season to another.

**FIGURE 2:**
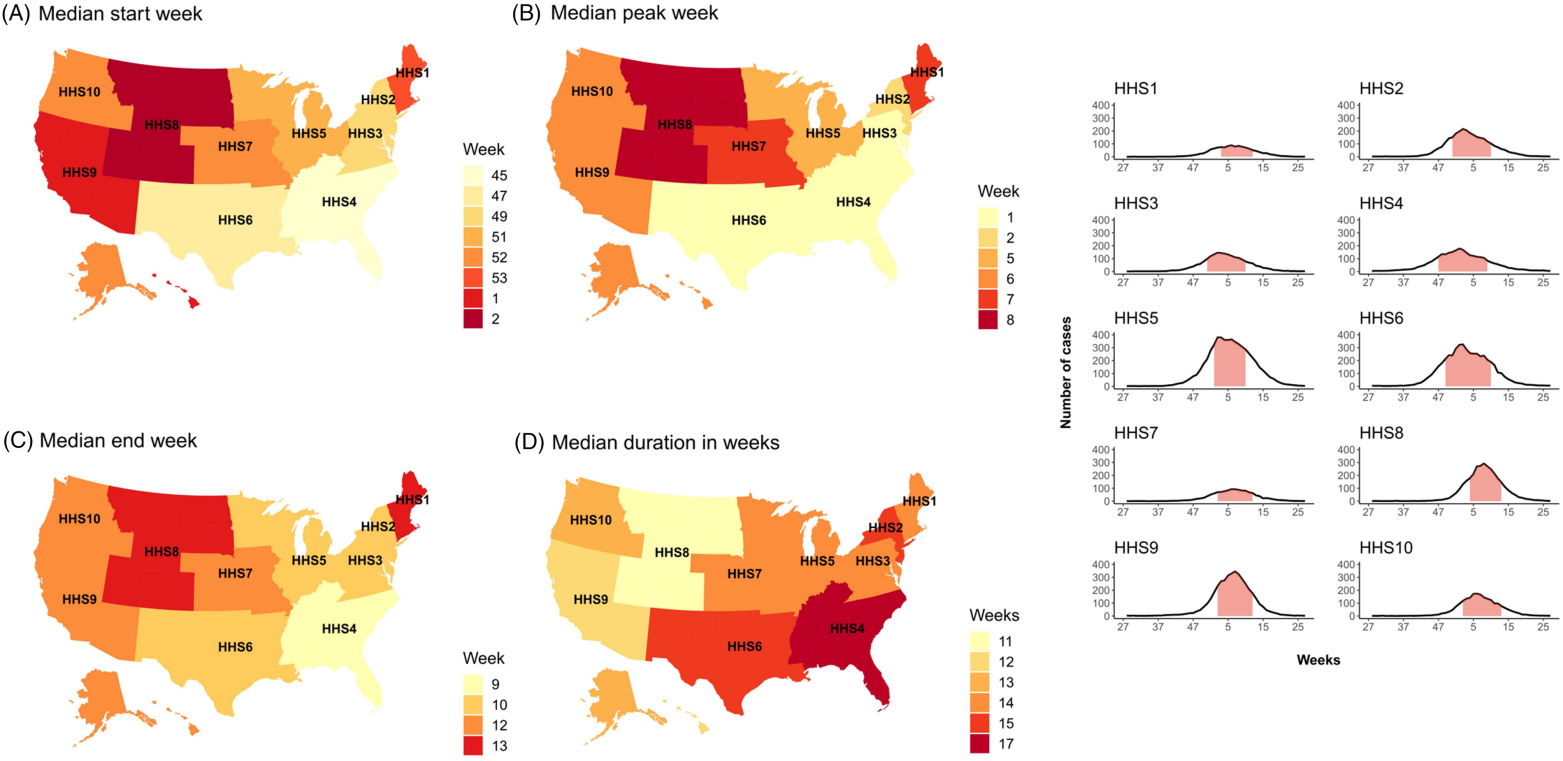
(A–D) RSV in the United States per region: Median seasonality and average curve. *Pink area under curve is median start–end epidemic; curve symbolized the average number of RSV cases each week*

The data in the United States were stratified for the 10 Health and Human Services (HHSs) departments. Though seasonality within each HHS was stable (Figures 1A and 2A–D), variation was found when comparing the different HHSs. The median season tended to start in HHS4 (Atlanta) in Week 45 and then moved to HHS6 (Dallas), HHS2 (NYC), and HHS3 (Philadelphia). The latest start of the RSV season was observed in Week 2 in HHS8 (Denver)—resulting in a 9‐week earlier start in the South East compared with the North West. The median duration of RSV activity ranged between 11 and 17 weeks, with the longest RSV seasons occurring in HHS4 (Atlanta) and the shortest in HHS8 (Denver).

### 3.2 (Sub)tropical climate zones

**FIGURE 3:**
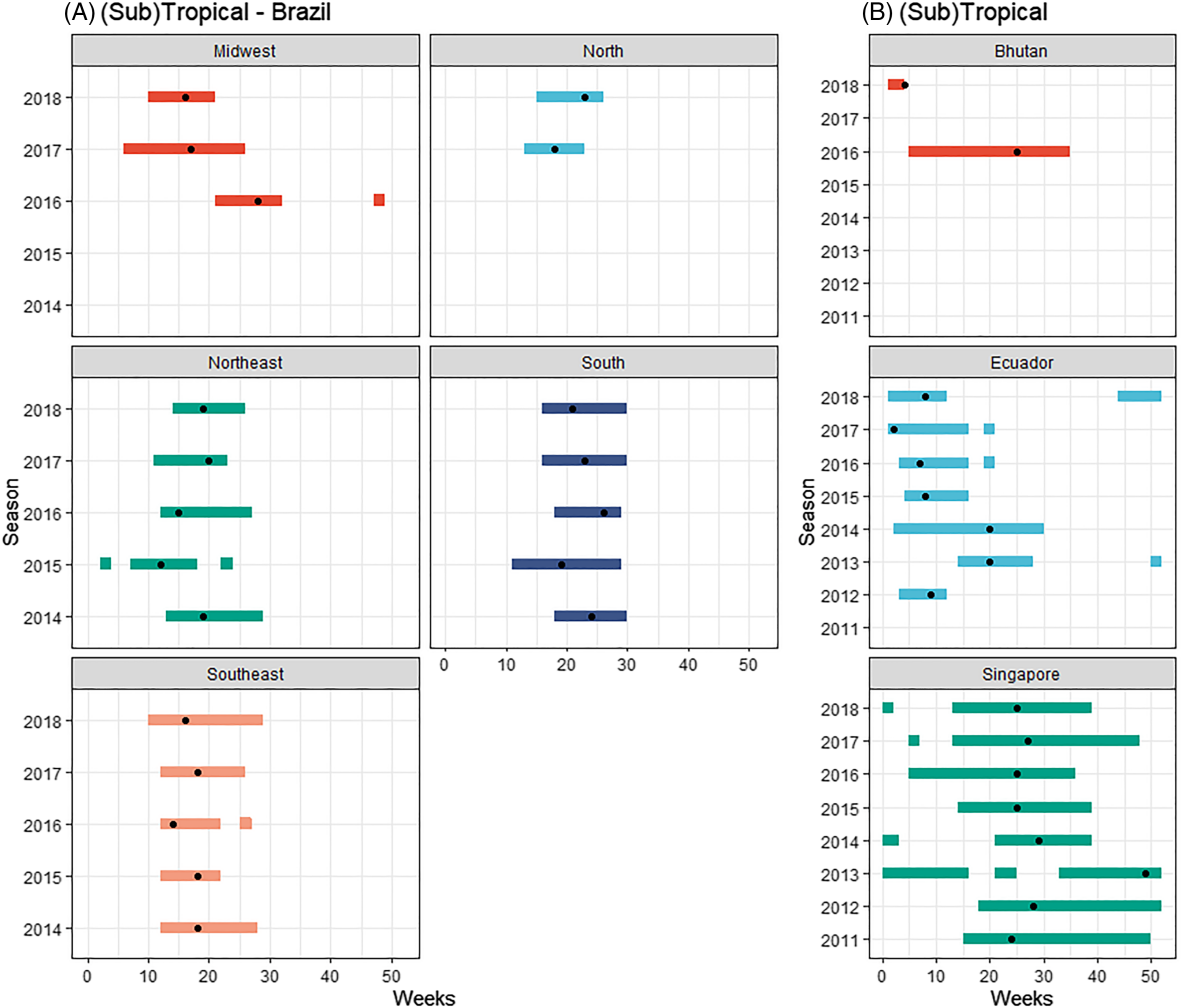
(A, B) RSV seasonality per country (or subnational level) and season in countries experiencing a (sub)tropical climate. *Dot indicates peak of the epidemic*

Less stability in seasonality was found across countries experiencing a (sub)tropical climate (Figure 3A,B). Singapore experienced an average of seven epidemic months. Except for the 2013 season, the epidemic peak fell between May and July; the other months regarded as “epidemic months” varied widely (Figure 3B). For Bhutan, two seasons with sufficient data were available (2016 and 2018). In 2018, the RSV cases were concentrated between Week 1 and Week 4, with 37% of the cases occurring in Week 4. In 2016, the cases were more distributed, resulting in a season lasting between Week 5 and Week 35. The RSV season in Ecuador had a median start in Week 3, an end in Week 16, and a duration of 13 weeks. Apart from the 2013 season, the epidemic consistently started in January with substantial variability in the end and duration of seasons. Several seasons showed epidemic weeks outside of the primary season. We also saw an increase in cases towards the end of 2018, possibly indicating an early start of the next epidemic.

**FIGURE 4:**
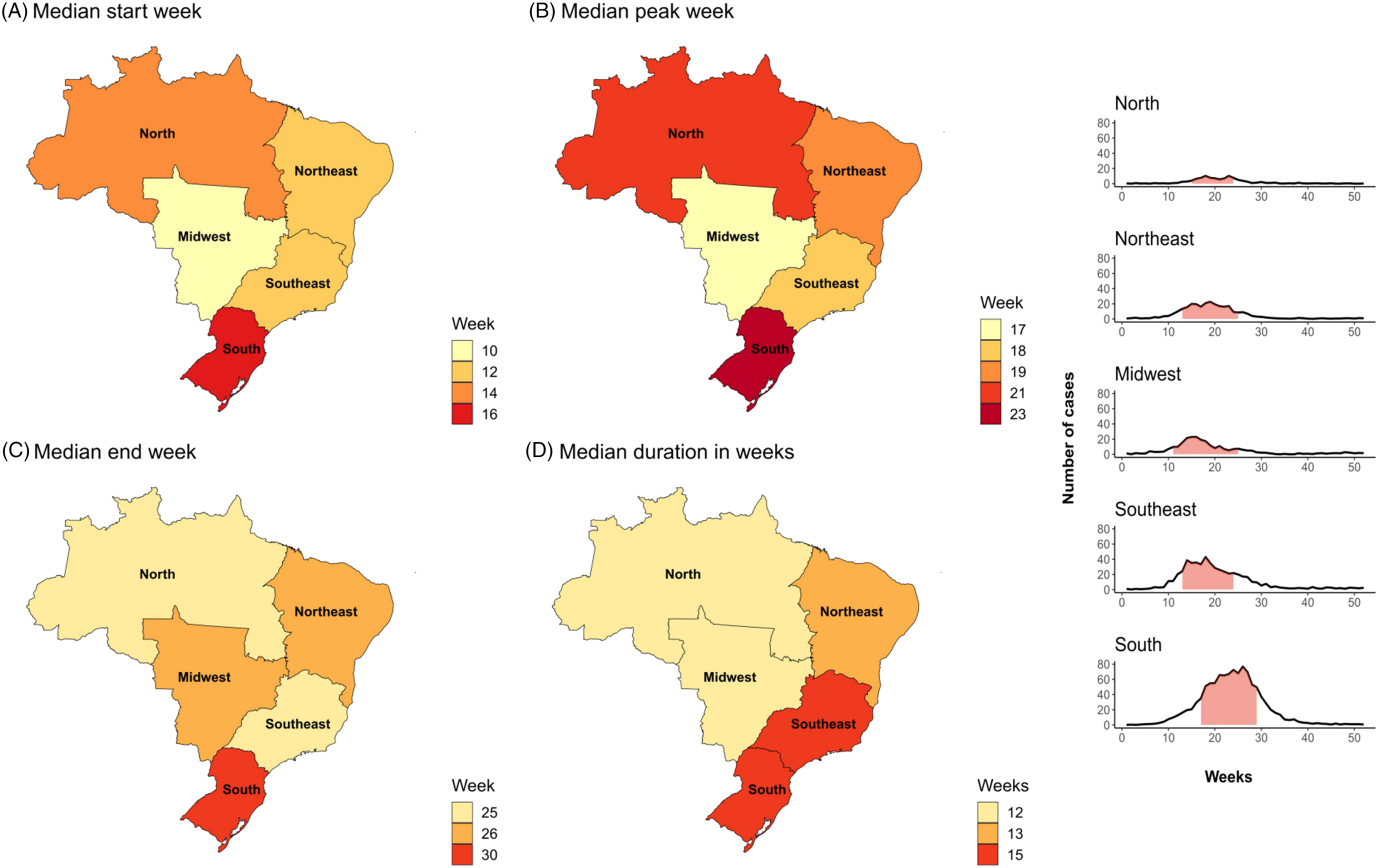
(A–D) RSV in Brazil per region: Median seasonality and average curve. *Shaded pink area indicated the median start and end of the epidemic; curve symbolized the average number of RSV cases each week*

On a national level, Brazil experienced an RSV season with a median start in Week 12, end in Week 27, and a median duration of 13 weeks. The Brazilian RSV surveillance data were by region (Figures 3A and 4A–D). The median RSV season started in Week 10 in the Midwest region and then moved to the North East and South East, the North, and ended in the South in Week 16.

### 3.3 Dominant subtypes and seasonality

**FIGURE 5:**
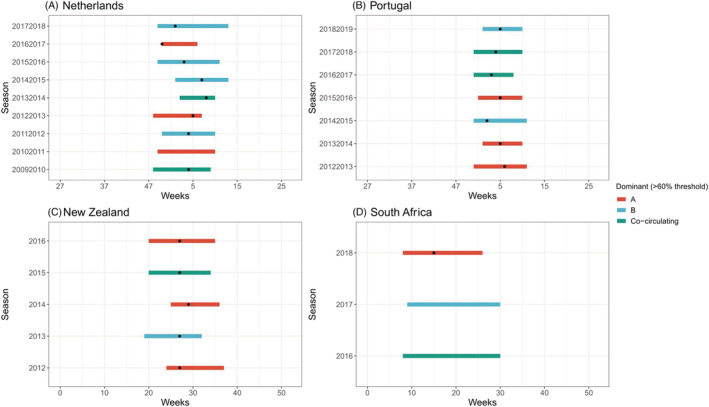
(A–D) Dominant RSV subtype (60% threshold) per season and country. *Dot indicates the peak of the epidemic*

**FIGURE 6:**
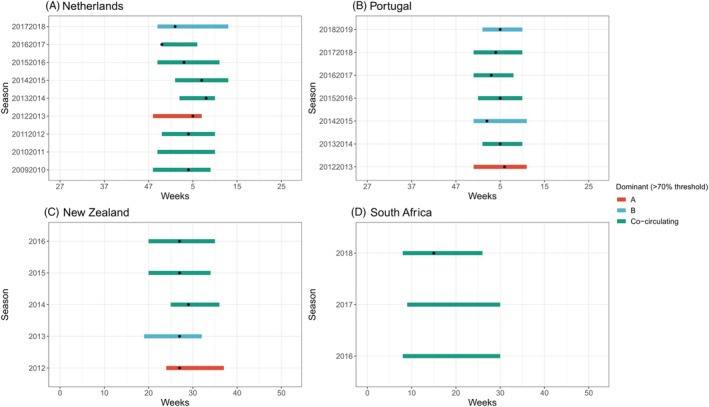
(A–D) Dominant RSV subtype (70% threshold) per season and country. *Dot indicates the peak of the epidemic*

Eighteen seasons in four different countries contained sufficient RSV subtype data to evaluate the impact of subtype dominant seasons on the timing of RSV seasons. The analysis was performed for both a 60% and 70% subtype dominant threshold. Using the >60% threshold, eight seasons were RSV B dominant and 10 were RSV A dominant (Figure 5A–D). We did not find substantial differences in the start of the epidemic when comparing RSV A or RSV B dominant seasons; however, RSV B seasons ended 1 week later and lasted 2 weeks longer. Neither of these results was significant (*p* = .80 and *p* = .07, respectively). The >70% threshold gave four RSV B and three RSV A dominant seasons (Figure 6A–D). No substantial differences in duration, start, or end of the epidemics were found.

## 4 DISCUSSION

This study analyzed RSV seasonality using a uniform method across a multicountry dataset of virologically confirmed surveillance data. Data from 12 countries around the world were available as part of the GERi study, which resulted in the inclusion of a total of 501 425 cases of RSV across 210 seasons (131 seasons of which are subnational). Our findings showed consistent, annual RSV epidemics in temperate climates during the winter months and less consistent epidemics in the (sub)tropics. However, not all our national assessments fit this pattern in a uniform manner, and our analysis showed that even subnational variation exists. These findings highlight the need for (sub)national RSV surveillance data to determine the optimal start and duration for prevention and control measures in large countries. Our findings suggest that the dominant RSV subtype has limited influence on the seasonality of RSV.

Having a better understanding of RSV epidemics is important to ensure the optimal timing of prevention and control measures. This is relevant as previous generalizations regarding the seasonality of influenza have resulted in suboptimal vaccination strategies,^24^ which would be especially problematic in the case of RSV where the administration of a prophylaxis is often more time sensitive or requires multiple doses throughout the RSV season (e.g., mAbs).^5^, ^25^ As such, increased awareness of the timing of RSV epidemics ensures that optimal levels of protection are achieved in a cost‐effective manner. In addition, better knowledge on the timing of RSV activity will allow health care providers to better prepare and manage limited resources, which are often stretched in the winter months, in an efficient manner.

Our findings largely confirm previous studies that have shown consistent, annual RSV epidemics in temperate climates during the winter months and less consistent epidemics in the (sub)tropics.^7^, ^12^, ^13^, ^14^, ^15^ In addition, studies have found associations between RSV seasonality and the latitude and longitude of a country. Most studies have found a positive correlation with latitude, ^8^, ^12^, ^13^, ^15^ as peak RSV activity generally occurs later in the year with increased latitude in both the Northern and Southern Hemispheres. One region where this is not the case is Europe, where three different studies have found contradictory results. One study found latitude to be positively associated with RSV activity, one study found the epidemic to move from several Northern European cities (e.g., Helsinki and Stockholm) to the South (as well as further North), and a third found no association.^8^, ^12^, ^13^ The latter study found RSV epidemics in the East to be later than in the West (i.e., a longitudinal association).^13^

The positive association between latitude and RSV seasonality is largely shown in our data, but several exceptions were noted. In the United States, RSV activity appeared to start in the most Southern region (HHS4) with a later start in higher latitudes (e.g., HHSs 1, 8, or 10). However, not all HHS regions fitted this pattern perfectly as exemplified by a relatively late start in HHS9 (San Francisco). In Europe, one would have expected our data to show the earliest RSV peak in Portugal, followed by Spain, the Czech Republic, and finally, the Netherlands—which was not the case in general. The longitudinal association outlined above could explain the later start of RSV epidemics in the Czech Republic compared with our results for other European countries. Another feature of RSV activity in some European countries has been a 2‐year periodicity in the form of an alternating start or size of the epidemic, which has been previously described in, for instance, Croatia.^12^ We did not observe this pattern in Portugal, Spain, or the Netherlands. Our analysis in the Czech Republic (five seasons) may indicate a 2‐year periodicity seasonality pattern, where at least the start of the epidemic alternates from 1 year to the next (early‐late‐early‐late). However, the timing of the RSV epidemic in the Netherlands, as previously described,^26^ did appear to show a different form of periodicity, defined by the authors as an amplitude‐like pattern.

In the Southern Hemisphere, our results were largely in line with the hypothesis that there is an association between RSV activity and latitude, as peak activity occurred first in (sub)tropical countries (Ecuador and Brazil) followed by temperate countries (Chile and New Zealand). One exception was South Africa, in the same hemisphere and climatic zone as Chile, where RSV epidemics tended to start substantially earlier (median start in Chile was Week 23 vs. Week 8 in South Africa). In addition, our findings showed that there appeared to be more variability in the end and thus duration of the epidemics in South Africa. Both factors are thought to be somewhat atypical for a country experiencing a temperate climate.^10^ One study found that this might be due to higher levels of precipitation and humidity during the months when RSV circulates.^9^

Several studies have explored the association between RSV activity and weather conditions, especially in the (sub)tropics where RSV activity appears less consistent.^9^, ^13^, ^27^ Such associations not only explain variation in the timing of RSV epidemics between countries but also season‐to‐season variation on a (sub)national level. These studies have suggested that RSV epidemics occur in conjunction with the humid and/or rainy season, which for countries located near the equator explains the tendency for year‐round or long‐lasting RSV epidemics.^7^, ^28^ The latter provides a potential explanation for the year‐round circulation of RSV that has been observed in Singapore. Similarly, a study conducted in the Netherlands showed how some differences in the timing of RSV epidemics from season to season could be explained by relative humidity as well as temperature variations.^27^ Though the association between low temperatures and RSV activity in temperate countries has been well documented, evidence regarding this association in the (sub)tropics has been conflicting.^28^

We were able to assess the timing of RSV epidemics on a subnational level for two countries: the United States and Brazil. On a national level, the United States, a temperate country in the Northern Hemisphere, experienced RSV epidemics typical for its location—with the majority of RSV activity occurring in the winter. However, on a subnational level, there was considerable seasonal variation, with the RSV season starting in the South East and then moving to the North West. The start of the RSV season differed by approximately 2 months. This pattern has been described by other studies^16^, ^29^; however, a clear explanation for the earlier start in Florida is unknown.^16^ Part of the explanation may lie in its climate. The way in which we have defined climate zones implies that Florida experiences a temperate climate based on its latitude; however, the South of Florida, the most populous area (e.g., Miami), is known to experience a tropical climate.^30^ On a national level, Brazil appears to fit the association with latitude on a national level. However, this was not the case on a subnational level, as seasons appeared to first start in the Midwest and the latest onset was experienced in the South (Figure 2A–D)—a pattern that largely overlaps with findings from a previous study.^31^ Results from both the United States and Brazil underline how national generalizations might misrepresent the epidemic activity of RSV in different parts of large and climatically diverse countries.

To the best of our knowledge, limited research has been done on the impact of the RSV subtype on the seasonality of RSV. One study found no such relation^26^ whereas one study in Beijing found an association for longer and earlier epidemics during RSV A dominant seasons.^19^ We did not find this association in our current and previous study,^20^ but more data are needed to validate this finding. As such, we would urge countries to increase the collection of subtype data as part of their RSV surveillance systems.

We have outlined how previous studies have sought clear predictors of RSV seasonality by examining its association with factors such as latitude, longitude, and the climate.^9^, ^12^, ^13^, ^15^ Though the timing of national RSV epidemics in the current analysis is largely in line with previous studies, there is no explanation for several outliers (e.g., South Africa, the late epidemic in California, and the patterns in Europe), and the literature appears contradictory in some cases. Neither the geographical location nor climatic factors is therefore deemed the universal and clear predictor of the timing of RSV epidemics.

We were able to compare the seasonal activity of RSV epidemics with previous studies by looking at the overall patterns, but comparing the exact metrics, such as the start or duration, is more complicated as differences exist in the methods used to estimate the metrics. This is exemplified by the CDC assessment of US RSV seasonality using the same dataset. The CDC estimates the average duration of national RSV epidemics to be 30 weeks^29^—whereas we have estimated it to be 14 weeks. In addition, two studies found that the duration of RSV epidemics in both hemispheres is about 5 or 6 months,^7^, ^15^ whereas we found an average duration of approximately 3 to 4 months, which is a closer approximation of a previous estimate by Li et al. (4.6 months) using the same AAP method.^13^ Our study highlights how the diversity in approaches used to estimate seasonality results in varying, and sometimes contradictory, outcomes, which underlines the need to harmonize seasonality estimation methods.

This study is one of the few studies that has used a multicountry dataset for the estimation of (sub)national RSV seasonality. The strength of the study is that it uses a diverse dataset spanning 12 different countries around the world. One method could therefore be applied to analyze RSV seasonality allowing for comparison between countries. A limitation of an analysis that compares seasonality across countries might be the discrepancies in surveillance systems as previously described,^22^ with factors such as case definitions and the surveillance setting potentially having implications on the estimation of RSV seasonality.^32^ Another limitation of our study is the lack of representation of several regions of the world as no data from countries in North Africa or the Middle East were included.

## 5 CONCLUSIONS

The majority of countries included in this analysis experience clear and stable RSV seasonality that is in line with previous studies. However, we came across countries in which RSV epidemics do not consistently fit this picture (e.g., South Africa or Singapore) or show considerable subnational variation (Brazil and the United States). National or even subnational data collection and analysis in large countries with diverse climates is important as regional or climatic generalizations of seasonality might misrepresent RSV epidemics. Additionally, we also found considerable variation when comparing our results with previously published estimates. The timing of epidemics appears to be unaffected by the dominant RSV subtype; however, more research is needed by assessing more seasons and a larger number of countries. The issue of RSV seasonality is important to efficiently implement prevention and control measures, and it is therefore critical to harmonize the methods used to define seasonality and gain a better understanding of RSV seasonality on a (sub)national level around the world.

## AUTHOR CONTRIBUTIONS

**Lisa Staadegaard:** Conceptualization; data curation; formal analysis; methodology; validation; visualization. **Saverio Caini:** Conceptualization; data curation; formal analysis; funding acquisition; validation. **Sonam Wangchuk:** Data curation; validation. **Binay Thapa:** Data curation; validation. **Walquiria Ferreira de Almeida:** Data curation; validation. **Felipe de Carvalho:** Data curation; validation. **Rodrigo Fasce:** Data curation; validation. **Patricia Bustos:** Data curation; validation. **Jan Kynčl:** Data curation; validation. **Ludmila Novakova:** Data curation; validation. **Alfredo Bruno:** Data curation; validation. **Doménica de Mora:** Data curation; validation. **Adam Meijer:** Data curation; validation. **Mariette Hooiveld:** Data curation; validation. **Q. Sue Huang:** Data curation; validation. **Tim Wood:** Data curation; validation. **Raquel Guiomar:** Data curation; validation. **Ana Paula Rodrigues:** Data curation; validation. **Vernon Lee:** Data curation; validation. **Li Wei Ang:** Data curation; validation. **Cheryl Cohen:** Data curation; validation. **Jocelyn Moyes:** Data curation; validation. **Amparo Larrauri:** Data curation; validation. **Concepción Delgado‐Sanz:** Data curation; validation. **Clarisse Demont:** Conceptualization; resources. **Mathieu Bangert:** Conceptualization; resources. **Michel Duckers:** Conceptualization; data curation; formal analysis; supervision. **Jojanneke van Summeren:** Conceptualization; data curation; formal analysis; methodology; validation. **W. John Paget:** Conceptualization; data curation; formal analysis; funding acquisition; supervision; validation.

### PEER REVIEW

The peer review history for this article is available at https://publons.com/publon/10.1111/irv.12885.

## Supporting information


**Table S1:** Summary of national seasonality, expressed as the start, end and duration of the season by hemisphere and climate zone.

## Data Availability

The data that support the findings of this study are available from the authors upon reasonable request.
