# Supplementary material for: Defining the seasonality of respiratory syncytial virus around the world: National and subnational surveillance data from 12 countries
Source: Influenza Other Respir Viruses. 2021 Jul 13;15(6):732–41. doi: 10.1111/irv.12885 (PMC8542954; doi:10.1111/irv.12885)
Supplement: Supplementary file 1 — Table S1: Summary of national seasonality, expressed as the start, end and duration of the season by hemisphere and climate zone. [file IRV-15-732-s001.docx]

**Supplement**

**Table 1: Summary of national seasonality, expressed as the start, end and duration of the season by hemisphere and climate zone.**

| Hemisphere | Climate zone | Country | Region | Seasons | Median Start week (range) | Median end week (range) | Median peak week (range) | Median duration in weeks (range) | Latitude (⁰)* | Longitude (⁰)* |
| --- | --- | --- | --- | --- | --- | --- | --- | --- | --- | --- |
| Northern | Temperate | Czech Republic | National | 2014/2015 - 2018/2019 | 3 (49 – 6) | 12 (10 – 18) | 8 (6 – 12) | 11 (10 – 14) | 50.08 | 14.44 |
|  |  | Netherlands | National | 2000/2001- 2018/2019 | 49 (45 – 52) | 8 (1 – 12) | 52 (50 – 8) | 11 (8 – 13) | 52.37 | 4.90 |
|  |  | Portugal | National | 2013/2014 - 2018/2019 | 51 (50 – 52) | 9 (7 – 10) | 4 (2 – 4) | 11 (10 – 12) | 38.72 | 9.14 |
|  |  | Spain | National | 2006/2007 - 2018/2019 | 48 (44 – 50) | 6 (5 – 8) | 1 (47 – 4) | 10 (9 – 13) | 40.42 | 3.70 |
|  |  | USA | National | 2006/2007 – 2018/2019 | 51 (40 – 5) | 11 (4 – 17) | 5 (47 – 11) | 14 (9 – 20) | 40.71 | 74.00 |
|  |  |  | HHS1 (ref: Boston) | 2009/2010 – 2018/2019 | 1 (48 – 5) | 12 (9 – 17) | 6 (51 – 9) | 14 (11 – 15) | 42.36 | 71.06 |
|  |  |  | HHS2 (ref: NYC) | 2008/2009 – 2018/2019 | 49 (46 – 1) | 10 (4 – 15) | 2 (52 – 10) | 15 (11 – 15) | 40.71 | 74.00 |
|  |  |  | HHS3 (ref: Philadelphia) | 2007/2008 – 2018/2019 | 49 (43 – 1) | 10 (7 – 12) | 1 (51 – 7) | 14 (12 – 18) | 39.95 | 75.16 |
|  |  |  | HHS4 (ref: Atlanta) | 2009/2010 – 2018/2019 | 45 (40 – 52) | 9 (6 – 13) | 1 (47 – 8) | 17 (14 – 20) | 33.75 | 84.39 |
|  |  |  | HHS5 (ref: Chicago) | 2006/2007 – 2018/2019 | 51 (45 – 4) | 10 (8 – 15) | 5 (52 – 10) | 14 (12 – 17) | 41.88 | 87.63 |
|  |  |  | HHS6 (ref: Dallas) | 2008/2009 – 2018/2019 | 47 (42 – 52) | 10 (6 – 11) | 1 (49 – 7) | 15 (11 – 20) | 32.78 | 96.80 |
|  |  |  | HHS7 (ref: Kansas City) | 2008/2009 – 2009/2010 & 2012/2013 – 2018/2019 | 52 (48 – 5) | 12 (11 – 15) | 7 (3 – 10) | 14 (9 – 16) | 39.10 | 94.58 |
|  |  |  | HHS8 (ref: Denver) | 2008/2009 – 2018/2019 | 2 (52 – 5) | 13 (9 – 17) | 8 (5 – 11) | 11 (10 – 13) | 39.74 | 104.99 |
|  |  |  | HHS9 (ref: San Francisco) | 2009/2010 – 2018/2019 | 52 (50 – 3) | 12 (9 - 17) | 6 (2 – 11) | 12 (11 – 17) | 37.77 | 122.42 |
|  |  |  | HHS10 (Seattle) | 2008/2009 – 2018/2019 | 52 (50 – 3) | 13 (10 – 16) | 6 (4 – 11) | 14 (12 – 17) | 47.61 | 122.33 |
|  | (Sub) Tropical | Bhutan^a^ | National | 2016 & 2018 | - | - | - | - | 27.47 | 89.63 |
|  |  | Singapore | National | 2011 – 2018 | Year round | Year round | Year round | Year round | 1.25 | 103.82 |
| Southern | (Sub) Tropical | Ecuador | National | 2012 – 2018 | 3 (1 – 14) | 16 (12 – 30) | 8 (2 – 20) | 13 (10 – 29) | 2.19 | 79.89 |
|  |  | Brazil | National | 2014 – 2018 | 12 (6 – 21) | 27 (18 – 32) | 19 (12 – 28) | 13 (11 – 21) | 23.55 | 46.63 |
|  |  | Brazil | North | 2017 – 2018 | 14 (13 – 15) | 25 ( 23 – 26) | 21 (18 – 23) | 12 (11 – 12) | 3.12 | 60.02 |
|  |  | Brazil | North East | 2014 – 2018 | 12 (7 – 14) | 26 (18 – 29) | 19 (12 – 20) | 13 (12 – 17) | 12.98 | 38.50 |
|  |  | Brazil | Midwest | 2016 – 2018 | 10 (6 – 21) | 26 (21 – 32) | 17 (16 – 28) | 12 (12 – 21) | 15.83 | 47.92 |
|  |  | Brazil | South east | 2014 – 2018 | 12 (10 – 12) | 25 (22 – 29) | 18 (14 – 18) | 15 (11 – 20) | 23.55 | 46.63 |
|  |  | Brazil | South | 2014 - 2018 | 16 (11 – 18) | 30 (29 – 30) | 23 (19 – 26) | 15 (12 – 19) | 25.43 | 49.27 |
|  | Temperate | South Africa | National | 2010 - 2018 | 8 (4 – 9) | 28 (16 – 47) | 13 (10 – 20) | 21 (12 – 41) | 33.92 | 18.42 |
|  |  | Chile | National | 2012 -2018 | 23 (21 – 25) | 35 (32 – 36) | 28 (26 – 29) | 12 (11 – 15) | 33.45 | 70.67 |
|  |  | New Zealand | National | 2012 - 2018 | 22 (19 – 25) | 36 (32 – 37) | 27 (27 – 31) | 11 (13 – 15) | 36.85 | 174.76 |

** latitude and longitude were determined on the basis of the most populous city in a given country*

*^a^ No median was calculated as only two seasons were included of which the timing was substantially different*
